# *Arabidopsis thaliana* endonuclease V is a ribonuclease specific for inosine-containing single-stranded RNA

**DOI:** 10.1098/rsob.210148

**Published:** 2021-10-20

**Authors:** Megumi Endo, Jung In Kim, Narumi Aoki Shioi, Shigenori Iwai, Isao Kuraoka

**Affiliations:** ^1^ Graduate School of Engineering Science, Osaka University, 1-3 Machikaneyama, Toyonaka, Osaka 560-8531, Japan; ^2^ Department of Chemistry, Faculty of Science, Fukuoka University, 8-19-1 Nanakuma, Jonan-ku, Fukuoka 814-0180, Japan

**Keywords:** endonuclease V, inosine, *Arabidopsis thaliana*, RNA damage, RNA editing

## Abstract

Endonuclease V is highly conserved, both structurally and functionally, from bacteria to humans, and it cleaves the deoxyinosine-containing double-stranded DNA in *Escherichia coli*, whereas in *Homo sapiens* it catalyses the inosine-containing single-stranded RNA. Thus, deoxyinosine and inosine are unexpectedly produced by the deamination reactions of adenine in DNA and RNA, respectively. Moreover, adenosine-to-inosine (A-to-I) RNA editing is carried out by adenosine deaminase acting on dsRNA (ADARs). We focused on *Arabidopsis thaliana* endonuclease V (AtEndoV) activity exhibiting variations in DNA or RNA substrate specificities. Since no ADAR was observed for A-to-I editing in *A. thaliana*, the possibility of inosine generation by A-to-I editing can be ruled out. Purified AtEndoV protein cleaved the second and third phosphodiester bonds, 3′ to inosine in single-strand RNA, at a low reaction temperature of 20–25°C, whereas the AtEndoV (Y100A) protein bearing a mutation in substrate recognition sites did not cleave these bonds. Furthermore, AtEndoV, similar to human EndoV, prefers RNA substrates over DNA substrates, and it could not cleave the inosine-containing double-stranded RNA. Thus, we propose the possibility that AtEndoV functions as an RNA substrate containing inosine induced by RNA damage, and not by A-to-I RNA editing *in vivo*.

## Graphical abstract

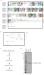

## 1. Introduction

The chemical structure of the base of DNA or RNA is subjected to deamination [1,2], and the loss of extra-cyclic amino groups due to deamination occurs spontaneously under physiological conditions via a hydrolysis reaction. Deamination of cytosine, adenine and guanine occasionally converts these bases to uracil, hypoxanthine and xanthine, respectively. Hypoxanthine in DNA and deoxyinosine are potentially mutagenic because they can base pair with cytosine during replication and generate A : T to G : C transition type mutations [3,4]. In *Escherichia coli*, deoxyinosine repair is initiated by endonuclease V (EndoV: EcEndoV from *E. coli*), which hydrolyses the second phosphodiester bond 3′ to hypoxanthine [5].

The deoxyinosine-repair mechanism, known as the alternative excision repair pathway using EcEndoV, has been elucidated [6,7]. After one side nicking by endonuclease V, the damaged nucleotide is removed by proofreading 3′–5′ exonuclease activity of DNA polymerase I (pol1), thereby generating a 2 nt gap. Eventually, pol1 fills this gap, and DNA ligase seals the nick. Therefore, the primary role of EcEndoV is to initiate the repair of deoxyinosine and prevent mutations.

By contrast, adenine in RNA is spontaneously hydrolysed to hypoxanthine. Inosine is a nucleoside in which hypoxanthine is attached to ribofuranose via a glycosidic bond. In higher organisms, three fundamental mechanisms contribute to the generation of inosine in RNA [8,9]. One mechanism is spontaneous or nitrosative deamination. The second mechanism includes the misincorporation of inosine triphosphate (ITP) into the transcript by RNA polymerases during transcription. Although ITP is an intermediate in the purine metabolism pathway and is rarely formed due to defects in purine nucleotide metabolism, it is removed by inosine triphosphatase from the cellular nucleotide pool [10,11]. Inosine generated in mRNA via these two processes is presumed to be potentially mutagenic because unexpected inosine in RNA is recognized as guanine during translation and can generate mutant proteins, thus changing the cellular phenotype. The third mechanism involves adenosine-to-inosine (A-to-I) RNA editing by adenosine deaminase acting on dsRNA (ADARs) [12–14]. Although the biologically significant function of ADARs is presumed to be pre-programming and site-specific deamination in mRNA, they may also exhibit functions in the duplex regions of non-coding RNAs, including microRNAs, small interfering RNAs and viral RNAs. A-to-I editing is the most common type of RNA editing in metazoan species and can modify the protein function, generate new proteins and alter gene regulation.

EndoV is highly conserved from bacteria to mammals; however, it differs in the substrate specificities. In *E. coli*, this enzyme activity has also been reported on DNA substrates containing apurinic/apyrimidinic site (AP site), urea, mismatches, hairpins, loops and pseudo-Y structures [15–17]. In *Thermotoga maritima* (Tma), TmaEndoV (Endo V from Tma) activity is observed for DNA substrates containing deoxyinosine, AP site, uracil or mismatches [18,19]. In eukaryotes, *Schizosaccharomyces pombe* (SpEndoV), invertebrate *Ciona interstinails* (CiEndoV), *Mus musculus* (MmEndoV) and *Homo sapiens* (HsEndoV) preferentially comprise a ribonuclease for single-stranded RNA substrates containing inosine [20]. The cleavage patterns of these nucleases were similar between bacteria and eukaryotes. HsEndoV co-localizes to the cytosol and nucleolus [21,22]; thus, it is unlikely to function in DNA repair, but may play a role in RNA processing, including A-to-I RNA editing.

The present study focuses on *A. thaliana* endonuclease V (AtEndoV) activity with variations in DNA or RNA substrate specificities. *A. thaliana* is an important model plant used for identifying genes and determining their functions, and moreover, the genomic sequence of *Arabidopsis* has been reported in the database [23,24]. In plants, RNA editing can be carried out for deamination of cytosine but not of adenosine because no ADAR is found for A-to-I editing in *A. thaliana*. Thus, we might rule out the possibility of the effects of inosine generation by A-to-I editing.

## 2. Material and methods

### 2.1. DNA and RNA substrates

21-mer ssDNA-containing deoxyinosine (ssDNA(dI)) substrates (5′-CTGTATGATGdIAGATGCTGAC-3′) and 21-mer ssRNA-containing inosine (ssRNA(I)) substrates (5′-CUGUAUGAUGIAGAUGCUGAC-3′) as well as the complementary strands of 21-mer RNA substrates (5′-GUCAGCAUCUUCAUCAUACAG-3′, 5′-GUCAGCAUCUCCAUCAUACAG-3′) were synthesized by FASMAC (Kanagawa, Japan) and purified using high-performance liquid chromatography. The oligonucleotides were 5′-phosphorylated using (γ-32P)-ATP (PerkinElmer Life Sciences, Waltham, MA) and T4 phosphoramidite kinase (TaKaRa, Shiga, Japan). To perform the cleavage assay for dsRNA(I) containing inosine, the ^32^P-labelled ssRNA(I) was annealed to the complementary 21-mer RNA substrate in an annealing buffer (10 mM Tris-HCl [pH 7.9], 50 mM NaCl, 10 mM MgCl and 1 mM DTT).

### 2.2. Proteins

RIKEN BRC, which is participating in the National BioResource Project of the MEXT/AMED, Japan, provided AtEndoV cDNA (RIKEN Arabidopsis full-length cDNA clone, resource number: pda13534, cDNA clone name: RAFL21-57-O06). AtEndoV cDNA containing a 6xHistidine-tag on the C-terminus was cloned into pGEX-6p-2 (GE Healthcare, Amersham Place, UK) and was mutagenized to generate mutant AtEndoV (Y100A) using PrimeSTAR Mutagenesis Basal Kit (TaKaRa, Shiga, Japan). The constructs were verified by DNA sequencing. *E. coli* C41(DE3) was used to express the recombinant protein. Thereafter, the recombinant protein was purified using DEAE Sepharose FF, TALON Metal Affinity Resin (TaKaRa) and Glutathione Sepharose 4 Fast Flow, following the manufacturer's recommendations; it was further analysed by 10% sodium dodecyl sulfate–polyacrylamide gel electrophoresis (SDS-PAGE) and eventually visualized by Coomassie Brilliant Blue staining. Protein concentrations were measured using a Bio-Rad Protein Assay Kit (Bio-Rad, Hercules, CA, USA).

### 2.3. Cleavage assays

Standard cleavage reaction mixtures (10 μl) comprised 4 nM of ^32^P-labelled oligonucleotide substrates and the indicated amount of AtEndoV or mutant AtEndoV (Y100A) in a reaction buffer containing 50 mM potassium acetate, 20 mM Tris-acetate (pH 7.9), 10 mM magnesium acetate and 1 mM dithiothreitol. The reactions were incubated for 30 min at each reaction temperature and then terminated with 10 μl of sequencing stop buffer (containing 98% deionized formamide, 20 mM EDTA, 0.025% bromophenol blue and 0.025% xylene cyanol). Thereafter, the fragments were separated on a 12.5% denaturing polyacrylamide gel. Next, the dried gel was analysed using a Fuji FLA-7000 Phosphor Imager (Fujifilm, Tokyo, Japan).

### 2.4. Surface plasmon resonance analysis

The binding activity of AtEndoV on ssDNA(dI) and ssRNA(I) was determined using the ProteOn XPR36 protein interaction array system (Bio-Rad Laboratories, Hercules, CA, USA) and one ProteOn NLC sensor chip coated with NeutrAvidin to immobilize biotinylated ligands. ProteOn PBS/Tween (0.005% Tween 20), pH 7.4, was used as the running buffer. Here, we did not use any divalent metal ion to avoid cleavage reactions. The biotinylated 4.55 21-mer ssDNA(dI) substrate (5′-CTGTATGATGdIAGATGCTGAC-3′) and 21-mer ssRNA(I) substrate (5′-CUGUAUGAUGIAGAUGCUGAC-3′) were injected for 331 s at 25 µl min^−1^ in the vertical direction. Thereafter, 1996 response units (RU) of 21-mer ssDNA(dI) and 1450 RU of 21-mer ssRNA(I) were coupled to the NLC sensor chip. AtEndoV was diluted to 500, 250, 125, 62.5 and 31.25 nM in 1 mM EDTA and PBS/Tween and then injected into the horizontal analyte channel with a contact time of 60 s, dissociation time of 300 s and flow rate of 25 µl min^−1^. All experiments were performed at 25°C.

## 3. Results and discussion

### 3.1. Alignment of the amino acid sequences of AtEndoV

**Figure 1.**
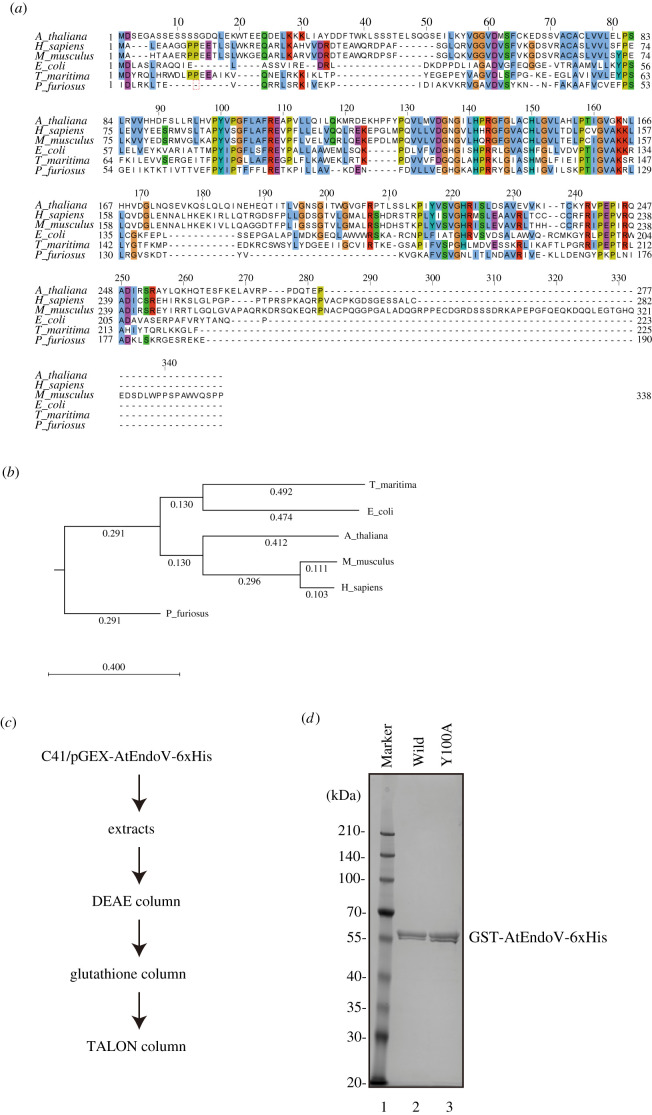
Purification of recombinant AtEndoV. (*a*) Alignment of the representative amino acid sequences of EndoV from *Arabidopsis thaliana* (NP_565382), humans (NP_775898), *Mus musculus* (NP_001158108), *Escherichia coli* (NP_290630), *Thermotoga maritima* (NP_229661) and *Pyrococcus furiosus* (WP_011012124). (*b*) Phylogenetic analysis of endonuclease V. A phylogenetic tree was constructed using the unweighted pair group method with arithmetic mean method based on the amino acid sequences of AtEndoV. (*c*) Experimental procedure for purification of AtEndoV. (*d*) Purification of recombinant AtEndoV and analysis on 10% SDS-PAGE gel stained with Coomassie Brilliant Blue. Lane 1, marker; lane 2, GST-AtEndoV(WT)-6xHis; lane 3, GST-AtEndoV(Y100A)-6xHis.

The open reading frame encoded a predicted 277 amino acid residue product, with a calculated molecular mass of 31 kDa. Alignment of the amino acid sequence against EcEndoV revealed identities = 67/194 (35%), positives = 105/194 (54%) and gaps = 19/194 (9%), whereas that against HsEndoV revealed identities = 89/244 (36%), positives = 105/244 (50%) and gaps = 19/244 (20%). Moreover, this alignment (figure 1*a*) indicates high conservation within the middle region containing catalytic residues. Analysis of the relationship between AtEndoV and other EndoVs presented a phylogenetic tree based on these nuclease amino acid sequences using the unweighted pair group method with arithmetic mean. This phylogenetic tree revealed that the aforementioned protein was more related to HsEndoV than to EcEndoV; however, by aligning the amino acid sequences, AtEndoV activity on DNA-containing deoxyinosine or on RNA-containing inosine substrates is hardly predictable.

### 3.2. Purification of the recombinant AtEndoV protein and its mutant protein

To examine the biochemical activities of the protein encoded by AtEndoV cDNA, we prepared recombinant AtEndoV with an N-terminal glutathione S-transferase (GST) and a C-terminal 6xHistidine tag in *E. coli*. The recombinant protein was purified via two-step affinity purification (figure 1*c*). The calculated relative molecular mass indicated that the purified AtEndoV bearing GST and 6xHis-tag migrated as a doublet band of approximately 57 kDa in SDS-PAGE (figure 1*d*). Furthermore, the mutant protein bearing an alanine substitution at Tyr100 in AtEndoV was also purified (figure 1*d*). Although a doublet of these recombinant proteins was observed due to the N-terminal GST-tag and C-terminal His-tag, it is unlikely to be a degradation product of AtEndoV. Presumably, the doublet occurred due to the protein structure of AtEndoV.

### 3.3. Endonuclease activity of AtEndoV

**Figure 2.**
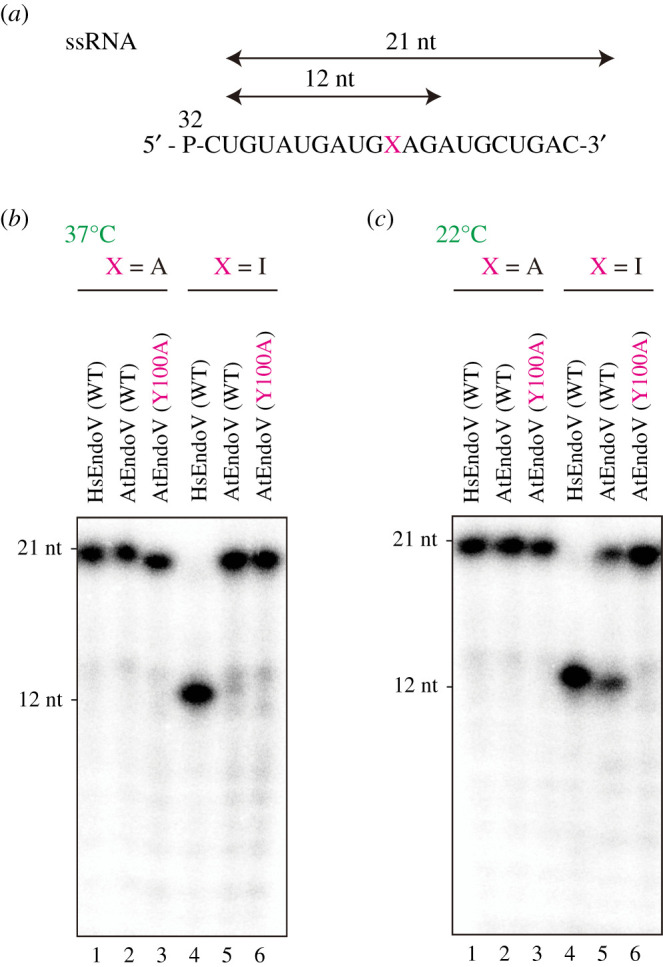
The effects of temperature on AtEndoV endonuclease activity. (*a*) ^32^P-labelled 21-mer ssRNA-containing adenosine (ssRNA(I)) (lanes 1–3) and inosine (lanes 4–6) in position X. (*b*) ^32^P-labelled ssRNA substrates were incubated with HsEndoV (150 nM; lanes 1 and 4) or AtEndoV (150 nM; lanes 2 and 5) or Y100A (150 nM; lanes 3 and 6) at 37°C (*b*) or 22°C (*c*) for 30 min. Fragments were separated on a 12.5% denaturing polyacrylamide gel.

**Figure 3.**
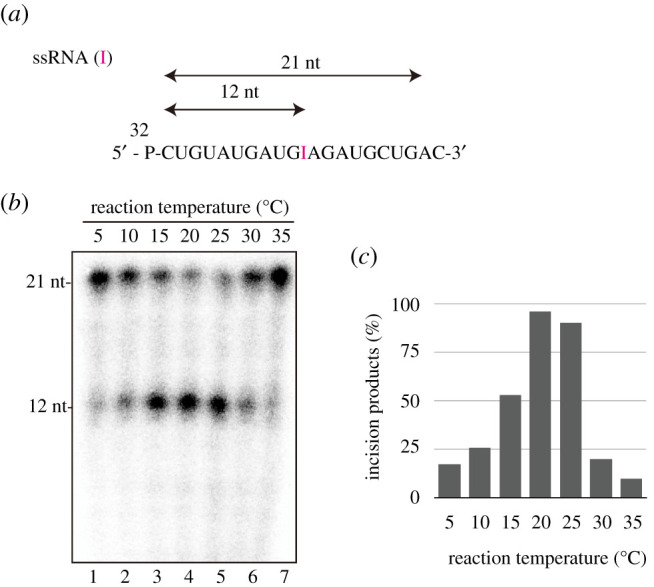
Temperature dependency of AtEndoV ribonuclease activity. (*a*) ^32^P-labelled 21-mer ssRNA-containing inosine (ssRNA(I)). (*b*) ^32^P-labelled ssRNA substrates were incubated with AtEndoV (150 nM; lanes 1–7) at the indicated temperatures for 30 min. (*c*) The right bar graph indicates the effect of incubation temperature on AtEndoV ribonuclease activity.

To analyse the ability of AtEndoV in cleaving the second and third phosphodiester bonds 3′ to inosine [22,25], which is known as inosine 3′ endonuclease activity in HsEndoV, a 21-mer ssRNA oligonucleotide containing adenosine or inosine at residue 11 was synthesized (figure 2*a*) and incubated with AtEndoV at 37°C. AtEndoV exhibited reduced activity; however, HsEndoV could cleave ssRNA(I) at 37°C, the optimum temperature of HsEndoV (figure 2*b*). Since the optimal temperature of *A. thaliana* is 22–23°C [23,24], the endonuclease activity of AtEndoV was performed at 22°C. AtEndoV, like HsEndoV, could cleave ssRNA(I) substrates, but it exhibited less activity (figure 2*c*, lane 5). The mutation Y100A, a substrate recognition site [20,26], resulted in proteins that could not exhibit the inherent nuclease activity under our experimental conditions (figure 2*c*, lane 6). AtEndoV cleaved ssRNA(I) substrates at 22°C, but was ineffective at 37°C. Therefore, we investigated the effect of temperature on ribonuclease activity. When the 21-mer ssRNA(I) substrates (figure 3*a*) were incubated with AtEndoV at the indicated temperature for 30 min, AtEndoV functioned effectively at 20°C or 25°C around its growth temperature (figure 3*b*, lanes 4 and 5, figure 3*c*). Thus, we observed the AtEndoV ribonuclease activity at 22°C.

**Figure 4.**
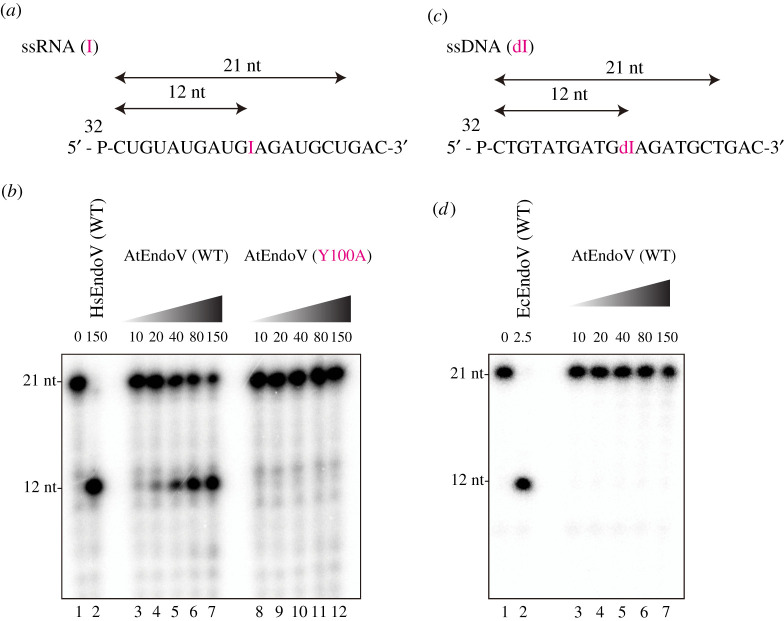
AtEndoV is a ribonuclease specific for ssRNA-containing inosine (ssRNA(I)). (*a*) ^32^P-labelled 21-mer ssRNA(I). (*b*) ^32^P-labelled ssRNA substrates were incubated with increasing concentrations of AtEndoV or Y100A (10, 20, 40, 80 and 150 nM in each group of five lanes) or HsEndoV (150 nM; figure 4*b*, lane 2). (*c*) ^32^P-labelled 21-mer ssDNA-containing deoxyinosine (ssDNA(dI)). (*d*) ^32^P-labelled ssDNA substrates were incubated with increasing concentrations of AtEndoV (10, 20, 40, 80 and 150 nM in each group of five lanes) or EcEndoV (2.5 nM; figure 4*d*, lane 2).

To confirm the AtEndoV activity, we investigated whether AtEndoV could cleave the ssRNA(I) substrate (figure 4*a*) in a protein concentration-dependent manner. As illustrated in figure 4*b*, AtEndoV (WT), like hEndoV (lane 2), could cleave the ssRNA(I) substrate (lanes 3–7); however, the mutant protein Y100A was catalytically impaired (lanes 8–12). The crystal structure of CiEndoV in complex with a hypoxanthine lesion [20] revealed that EndoVs have a conserved wedge motif that plays a pivotal role in deforming the substrate and flipping out the hypoxanthine. Y100 is a component of the wedge motif that was previously identified; therefore, Y100A might not cleave substrates.

**Figure 5.**
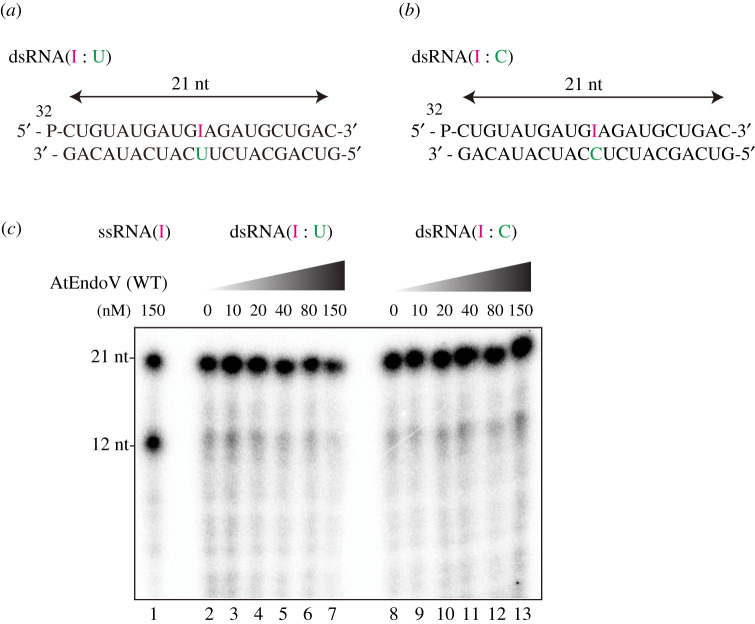
No cleavage of AtEndoV in inosine-containing dsRNA. (*a*) ^32^P-labelled 21-mer dsRNA-containing inosine paired with uridine. (*b*) ^32^P-labelled 21-mer dsRNA-containing inosine paired with cytidine. (*c*) AtEndoVs were incubated with ssRNA-containing inosine (ssRNA(I)) (AtEndoV: 150 nM; lanes 1), dsRNA-containing inosine paired with uridine (AtEndoV: 0, 10, 20, 40, 80 and 150 nM; lanes 2–7) or dsRNA-containing inosine paired with cytidine (AtEndoV: 0, 10, 20, 40, 80 and 150 nM; lanes 8–13).

While mammalian EndoVs exhibit high cleavage efficiency only for ssRNA(I) substrates, bacterial EndoVs could cleave both deoxyinosine-containing DNA and inosine-containing RNA substrates [8,20]; therefore, the purified AtEndoVs were further assayed to observe the endonuclease activity on ssDNA(I) (figure 4*c*) and dsDNA-containing I : U or I : C pairings (electronic supplementary material, figure 1Sa and 1Sb). A 21-mer ssDNA oligonucleotide containing deoxyinosine residue 11 was synthesized and incubated with AtEndoV at the optimal temperature of 22°C. Moreover, AtEndoV did not exhibit any nuclease activity on the ssDNA and dsDNA substrates under our experimental conditions (figure 4*d*; electronic supplementary material, figure 1Sa and 1Sb), thereby indicating that AtEndoV is not nuclease specific for deoxyinosine-containing DNA. To further investigate the ribonuclease activity of AtEndoV, we incubated AtEndoV with dsRNA containing inosine. While AtEndoV could cleave the ssRNA substrates in a protein concentration-dependent manner (figure 4*b*), the enzyme could generate any specific products in reactions with dsRNA-containing I : U (figure 5*a*) or I : C (figure 5*b*) pairings. The results of this study imply that AtEndoV exhibits ribonuclease specific activity towards ssRNA(I).

### 3.4. Atendov binds ssRNA with higher affinity than ssDNA

In the cleavage assay, AtEndoV preferentially cleaved ssRNA(I) substrates to ssDNA(dI). Moreover, to compare the binding activity of AtEndoV to ssRNA(I) or ssDNA(dI), we used the ProteOn XPR36 protein interaction array system. This system is based on surface plasmon resonance and can provide valuable information on the binding affinity and kinetics. Increasing amounts of AtEndoV, wild-type and catalytically inactive Y100A mutants were injected on a sensor chip containing either ssRNA(I) or ssDNA(dI). Certain parameters, including on-rate (*K*_on_(1/Ms)), off-rate (*K*_off_(1/s)) and equilibrium dissociation constant (*K*_D_ (1/M) = *K*_off_ *K*_on_^−1^) were determined (table 1). These results indicated that AtEndoV binds ssRNA(I) with a higher affinity than ssDNA(I). Furthermore, the catalytically inactive Y100A mutant exhibited a lower affinity than the wild-type. As previously reported, the results indicate that the wedge motif is involved in cleavage activity for recognizing hypoxanthine, and not for DNA or DNA binding.

**Table 1.** Kinetic parameters of *Arabidopsis thaliana* endonuclease V for ssRNA-containing inosine (ssRNA(I)) or ssDNA-containing deoxyinosine (ssDNA(dI)).

	substrate	*K*_on_ (1/Ms)	*K*_off_ (1/s)	*K*_D_ (M)
AtEndoV (WT)	ssRNA (I)	4.6 × 10^7^	3.4 × 10^−2^	2.0 × 10^−9^
	ssDNA (dI)	1.1 × 10^4^	2.6 × 10^−3^	2.2 × 10^−4^
AtEndoV (Y100A)	ssRNA (I)	6.3 × 10^3^	1.0 × 10^−3^	1.2 × 10^−7^
	ssDNA (dI)	3.7 × 10^3^	2.0 × 10^−3^	1.6 × 10^−4^

### 3.5. Features of AtEndoV

In the present study, we identified and characterized a novel plant endonuclease V-coding protein from *A. thaliana*. Analysis of AtEndoV nuclease activity revealed that AtEndoV cleaves ssRNA(I) at the second phosphodiester bond 3′ to inosine. We further investigated the effect of temperature on ribonuclease activity. AtEndoV effectively functioned around its growth temperature. This ribonuclease activity was similar to that of HsEndoV; however, the AtEndoV nuclease activity was low. While bacterial EndoVs can cleave DNA and RNA substrates [20], AtEndoV cannot cleave deoxyinonsine-containing DNA. Thus, Tyr100, an amino acid that constitutes a wedge motif on the protein surface, is crucial for ribonuclease activity. This observation implies that even though there seems to be a difference in the substrate specificity of EndoVs from bacteria to mammals, the mechanism of substrate recognition might be conserved to a certain degree.

Interestingly, so far, since ADARs were not found in *A. thaliana* or plants, at least A-to-I RNA editing by these enzymes might not exist [12]. Therefore, inosine in RNA is generated by spontaneous or nitrosative deamination or by incorporating ITP during transcription, and these reactions might be unexpected (RNA damage), unlike the ADAR reaction for RNA editing. Furthermore, a possible off-target reaction by adenosine deaminase acting on tRNA deaminates adenosine to inosine on tRNA. We speculate that AtEndoV functions in unexpected RNA damage, but not the scheduled RNA editing, and that other EndoVs bearing ribonuclease activity (e.g. SpEndoV, CiEndoV, MmEndoV and HsEndoV) might reveal similar results. However, we could not rule out the possibility that enzymes non-homologous to ADARs might have A-to-I RNA editing function in plants.

## Data accessibility

This article has no additional data.
